# Quantifying and correcting for tail vein extravasation in small animal PET scans in cancer research: is there an impact on therapy assessment?

**DOI:** 10.1186/s13550-015-0141-z

**Published:** 2015-11-05

**Authors:** Charline Lasnon, Audrey Emmanuelle Dugué, Mélanie Briand, Soizic Dutoit, Nicolas Aide

**Affiliations:** BioTICLA unit, UMR INSERM 1199, François Baclesse Cancer Centre, UNICAEN, Caen, France; Normandie University Caen, Caen, France; Biostatistics and Clinical Research Department, François Baclesse Cancer Centre, Caen, France; Nuclear Medicine Department, Caen University Hospital, Avenue Côte de Nacre, 14000 Caen, France

**Keywords:** Small animal PET, Cancer research, Therapy assessment, Tail vein injection, Extravasation, Quantification

## Abstract

**Background:**

Tail vein injection under short anesthesia is the most commonly used route for administering radiopharmaceuticals. However, the small caliber of the vein in rodents may lead to tracer extravasation and thereby compromise quantitative accuracy of PET. We aimed to evaluate a method for correction of interstitial radiotracer leakage in the context of pre-clinical therapeutic response assessment.

**Methods:**

In two separate studies involving 16 nude rats, a model of human ovarian cancer was xenografted and each was treated with a Phosphoinositide 3-kinase/mammalian target of rapamycin inhibitor or used as a control. Tracer injections were performed via the tail vein by a single operator. Two observers qualitatively evaluated the resulting images and if appropriate drew a volume of interest (VOI) over the injection site to record extravasated activities. Uncorrected and corrected tumors’ mean standardized uptake value (SUV)_mean_ was computed (corrected injected activity = calibrated activity − decay corrected residual syringe activity − decay corrected tail extravasated activity). Molecular analyses were taken as a gold standard. The frequency and magnitude of extravasation were analyzed, as well as the inter-observer agreement and the impact of the correction method on tumor uptake quantification.

**Results:**

Extravasation never exceeded 20 % of the injected dose but occurred in more than 50 % of injections. It was independent of groups of animals and protocol time points with *p* values of 1.00 and 0.61, respectively, in the first experiment and 0.47 and 0.13, respectively, in the second experiment. There was a good inter-observer agreement for qualitative analysis (kappa = 0.72) and a moderate agreement when using quantitative analysis (*ρ*_*c*_= 0.94). In both experiments, there was significant difference between uncorrected and corrected SUV_mean_. Despite this significant difference, mean percent differences between uncorrected and corrected SUVmean in the first and the second experiments were -3.61 and -1.78, respectively. Concerning therapy assessment, in both experiments, significant differences in median %SUV_mean_ between control and treated groups were observed over all time points with either uncorrected and corrected data (*p* < 0.05).

**Conclusions:**

Although extravasation is common and can be reproducibly corrected, this is probably not required for validation of response to drugs that induce large SUV changes. However, further studies are required to evaluate the impact of extravasation in situations where less marked metabolic responses are observed or important extravasations occur.

## Background

In cancer research, ^18^fluorodeoxyglucose small animal positron emission tomography/computed tomography (^18^FDG SA-PET/CT) is a recognized non-invasive tool for in vivo assessment of therapeutic response to novel therapies [1–4]. To this end, accurate quantitative values, such as standardized uptake values (SUVs), are mandatory. However, there are numerous confounding factors, including the mode of tracer administration.

Tail vein injection (with or without an intravenous catheter) under short anesthesia is the most commonly used route for administering radiopharmaceuticals. The small caliber of the vein in rodents may, however, predispose to tracer extravasation into the interstitium. Other validated administration routes are available, including retro-orbital and intraperitoneal injection [5, 6]. The main limitation of the first technique relates to restrictions imposed by many animal care committees. The second type of injection has several advantages. It is reproducible in longitudinal studies and can be performed on conscious animals, thereby reducing stress levels. However, a risk of failure remains (intra-digestive injection, [7, 8]), and it has not been validated in animals bearing intraperitoneal tumors in which peritoneal tracer absorption could be impaired. Moreover, even though delayed images (60 min) have been shown to be equivalent to intravenous injection [9–11], retro-orbital and intraperitoneal injections do not allow dynamic imaging [5].

Methods for measuring tail vein injection failure quantitatively have been developed [12, 13]. As far as we know, however, the impact of extravasation on quantitative measures of tumor uptake and whether these can be reliably corrected by estimation of extravasated activity has not been evaluated in rats. Therefore, we aimed to evaluate the impact of such a correction method in the context of therapeutic response assessment of a dual Phosphoinositide 3-kinase/mammalian target of rapamycin inhibitor (BEZ-235) on a chemoresistant model of human ovarian cancer xenografted in nude rats.

## Methods

### Animal model

The regional Ethics Committee granted approval to conduct this study (no. N/02-10-09/18/10-12). All procedures performed in this study involving animals were in accordance with the ethical standards of the regional research committee and with the 1964 Helsinki Declaration and its later amendments.

In two experiments, 16 4-week-old female nude rats (Harlan Laboratories, Indianapolis, IN, USA) bearing subcutaneous SKOV3 human ovarian tumors were used. Ten rats were treated with BEZ-235, a dual Phosphoinositide 3-kinase/mammalian target of rapamycin inhibitor (Selleck Chemicals, Houston, TX, USA) from day 0 to day 3, with treatment being discontinued on day 4. Six rats were used as controls. Each animal had four tumors: two in the shoulders and two in the upper thighs, providing 64 lesions for evaluation.

In the first experiment, five rats underwent PET imaging on days 0, 3, and 7. This cohort consisted of two groups: untreated controls (*n* = 2) and treated rats (*n* = 3). A second cohort was treated at the same time points and used for molecular analysis. In the second experiment, a cohort of 11 animals, consisting of untreated controls (*n* = 7) and rats receiving BEZ-235 (*n* = 4), was used for both PET imaging and molecular analysis. For the latter aim, one rat each from the treated and untreated groups was sacrificed on days 0, 3, and 4, and all remaining animals were sacrificed after the last PET examination had been performed on day 5. For general anesthesia, heated inhaled isoflurane was administered with an anesthesia device dedicated to small animals (Minerve, France).

Molecular analyses were performed in both experiments, including cell proliferation assessment (Ki-67 immunostaining), and Phosphoinositide 3-kinase/mammalian target of rapamycin target expression studies (p4E-BP1 immunostaining), which were taken as gold standards for therapeutic assessment in the present work.

### Cross-calibration

A cross-calibration among the SA-PET/CT system and the dose calibrator was performed. A 10-MBq ^18^F-FDG solution (as assessed by the dose calibrator) was used to fill a vial of exact known volume, which resulted in a solution of known concentration. This solution was used to fill a cylinder phantom that was scanned for 20 min on the SA-PET/CT scanner. A large volume of interest (VOI) was used to determine the mean activity concentration as assessed by the SA-PET/CT scanner. Cross-calibration factors were then derived and used to synchronize counts/measurements for the two pieces of equipment.

### SA-PET/CT acquisitions, reconstructions, and analysis

Animals were kept fasting for 6 h. As detailed above, SA-PET/CT (Inveon system, Siemens Medical Solution, Knoxville, TN, USA) examinations were performed on days 0, 3, and 7 in the first experiment and days 0, 3, 4, and 5 in the second experiment. The same individual (NA, with a 15-year experience in tail vein injection) performed tracer injections. Injections were performed intravenously through the tail vein, under general anesthesia, using a 29-gauge needle. Injected volume was always kept below 0.4 mL. The average calibrated activities were 38 ± 7 and 39 ± 5 MBq with uptake times of 91 ± 8 and 105 ± 14 min following injection, respectively, for the first and second experiments. Animals were imaged in prone position with the tail positioned on their right side. Reconstructions were performed using a NEMA NU 4-Optimized Maximum A Posteriori (MAP) Reconstruction [14] with scatter and attenuation corrections.

### SA-PET/CT analysis

**Fig. 1:**
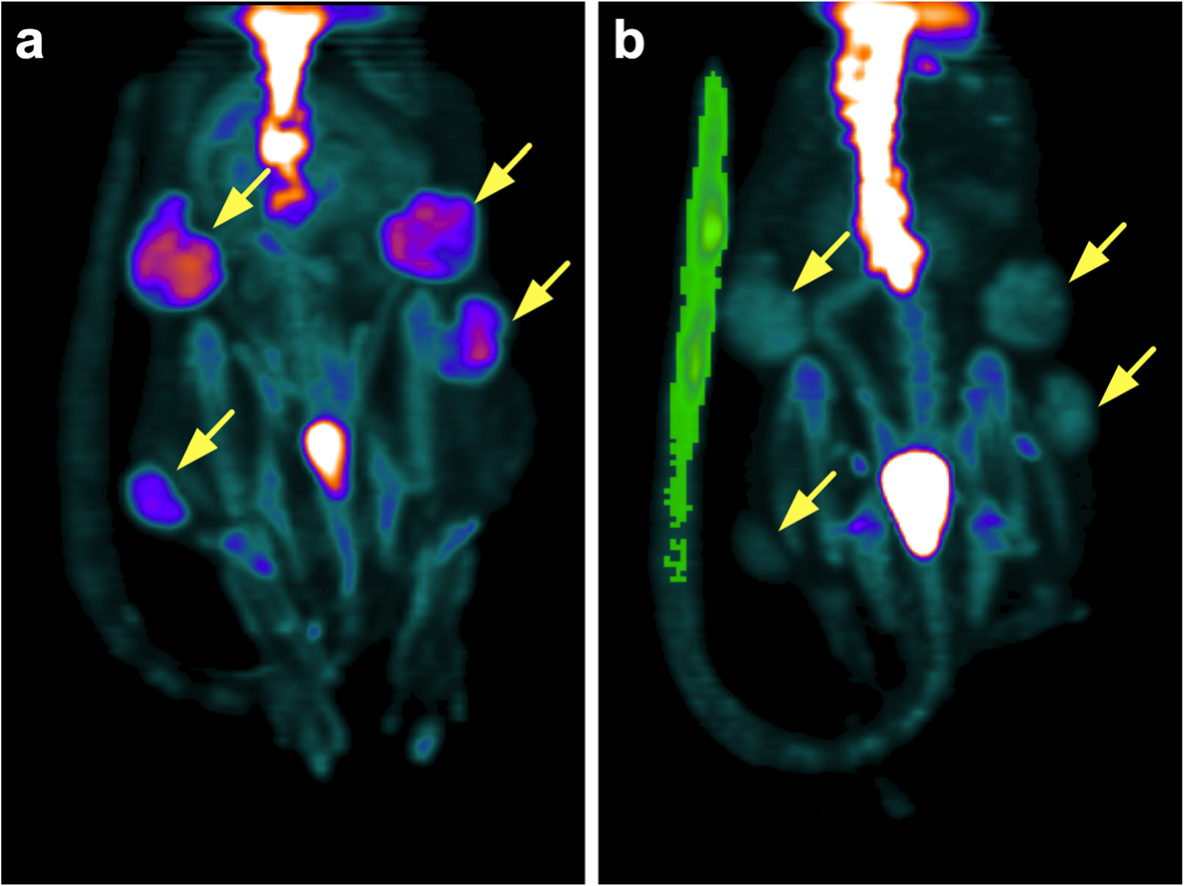
Representative images in animals with and without misinjection. Maximum intensity projection (MIP) visualization of the small animal positron emission tomography/computed tomography (SA-PET/CT) reconstruction for a treated rat in the second experiment without tracer extravasation on day 0 (**a**) and with tracer extravasation on day 3 (**b**). On day 3, a volume of interest (VOI) region was manually drawn and a visually adapted. Isocontour was applied (displayed in *green*) to quantify and correct for tail vein extravasation. *Yellow arrows* show the subcutaneous tumors

Intravenous injections were qualitatively evaluated from reconstructed images. When the observer concluded to the presence of extravasation, a three-dimensional volume of interest (VOI) with a visually adapted isocontour was drawn over the tail injection site (Fig. 1). Activity in the tail (Bq and Bq/cc) was recorded. Two independent observers made these analyses to determine the inter-observer variability. The activity of tail vein injection extravasation was corrected for decay assuming that there was no interstitial absorption of the tracer in the tail between the injection and the imaging session. Corrected injected activity was calculated by the following formula:

$$ \begin{array}{l}\mathrm{Corrected}\ \mathrm{injected}\ \mathrm{activity}=\\ {}\mathrm{calibrated}\ \mathrm{activity}-\mathrm{decay}\ \mathrm{corrected}\ \mathrm{residual}\ \mathrm{syringe}\ \mathrm{activity}-\mathrm{decay}\ \mathrm{corrected}\ \mathrm{tail}\ \mathrm{extravasated}\ \mathrm{activity}\end{array} $$

For therapeutic response assessment in each experiment, a cylindrical VOI was drawn over each tumor. The uncorrected and corrected mean pixel values were extracted for each VOI and SUV_mean_ were computed as follows, assuming a 1 g/mL density:

$$ \mathrm{S}\mathrm{U}\mathrm{V}=\frac{\mathrm{tumor}\ \mathrm{activity}\kern0.5em \left(\frac{\mathrm{Bq}}{\mathrm{mL}}\right) \times \mathrm{body}\ \mathrm{weight}\kern0.5em \left(\mathrm{g}\right)}{\mathrm{injected}\ \mathrm{dose}\ \left(\mathrm{Bq}\right)} $$

Data were processed with a dedicated Siemens station (Inveon Research Workplace 2.2, Siemens Molecular Imaging, Knoxville, TN, USA).

### Statistical analysis

These analyses aimed to determine (1) if tail vein extravasation corrected data performed better than uncorrected data for predicting target inhibition and (2) if there was inter-observer variability in the tail vein injection correction process. Statistical analyses were performed on a per-lesion basis without taking into account intra-rat correlation. Fisher’s exact probability tests, with the Freeman–Halton extension when necessary [15], were performed to test if the occurrence of tail vein extravasation was different according to groups (treated or not) and protocol time points. Inter-observer agreement was assessed by means of Cohen’s kappa coefficients (qualitative analysis) and Lin’s concordance coefficient (quantitative analysis). Tail vein activity measurements (Bq) of observers A and B were compared using Bland–Altman method comparisons. Wilcoxon tests and Bland–Altman plots were used for paired comparisons of corrected and uncorrected data amongst treated and control groups. To assess the ability to discriminate between control and treated groups, relative SUV_mean_ values of day 0 for each group were compared over the different time points (day 3, 4, 5, and 7), for corrected and uncorrected data, using Mann–Whitney tests. For each test, the alpha risk was set at 0.05. Statistical analysis, graphs, and plots were performed with GraphPad Prism version 5.0 for Mac (GraphPad Software, La Jolla, CA, USA; www.graphpad.com).

## Results

### Tail vein extravasation frequency and magnitude

Both observers recorded that extravasation was visible in at least 50 % of injections. According to observer A, tail vein extravasation occurred in 92.3 and 59.4 % of SA-PET/CT examinations in the first and second experiments, respectively. In the first experiment, the percentage of injected activity remaining in the tail vein never exceeded 20 %, with a maximum of 16.9 % in a control rat on day 0.

According to observer B, tail vein extravasation occurred in 69.2 and 50.0 % of SA-PET/CT examinations in the first and second experiments, respectively. The maximum tail vein remaining activity was 14.8 % and was observed in the same animal described above.

**Fig. 2:**
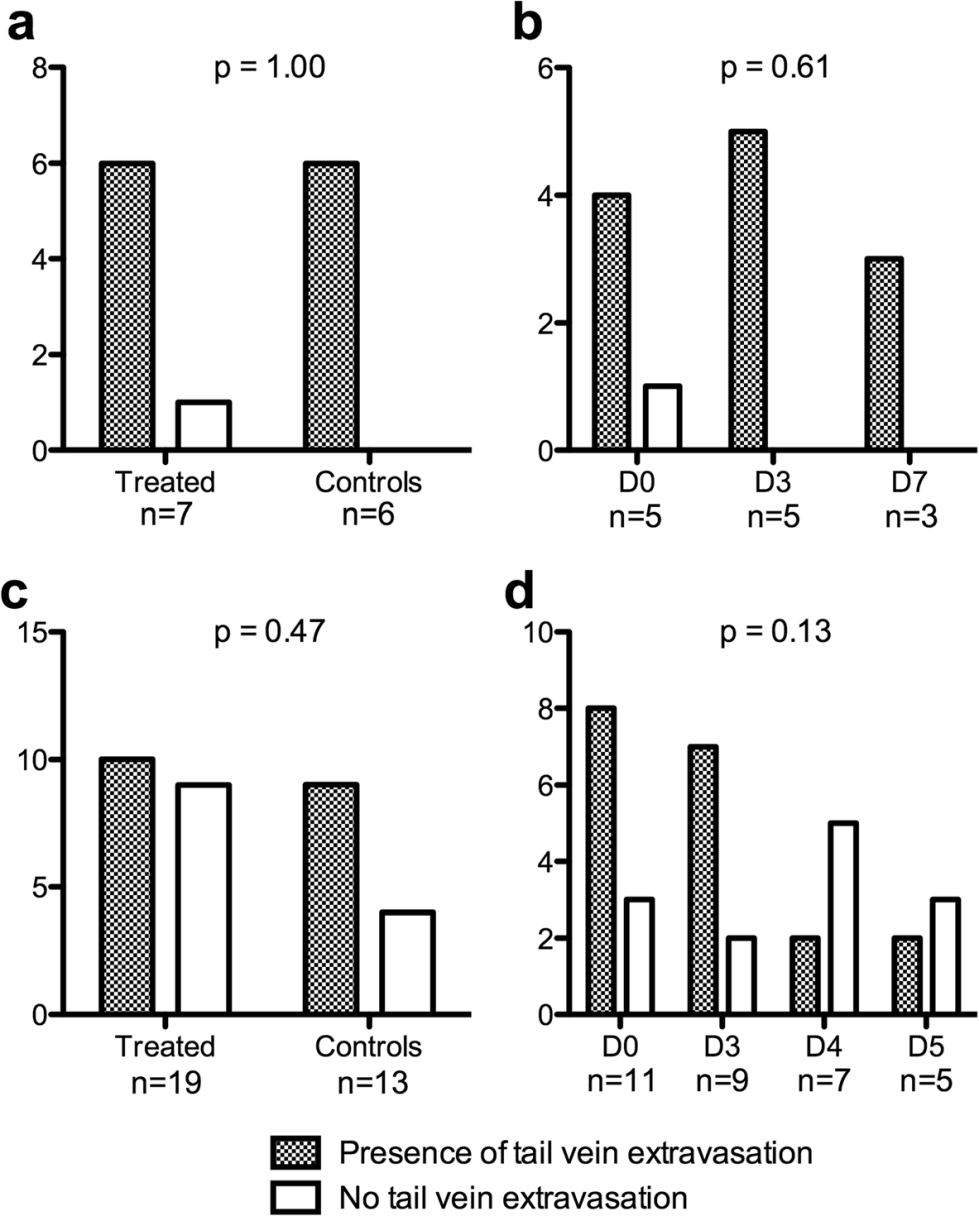
Frequency of tail vein extravasations depending on group and protocol time points. Panels **a** and **b** display the first experiment results and panels **c** and **d** display the second experiment results. For groups and protocol time point studies, only data of observer A is used. Of note, only three animals were scanned on day 7 because two treated rats had died from treatment toxicity

Ranges of tail vein extravasation activity values and their frequency are described in Table 1 for both observers. As shown in Fig. 2, the extravasation rate was independent of groups and protocol time points in the first and second experiments.

**Table 1:** Frequency and ranges of tail vein extravasation activity values

		Percentage of injected dose remaining in tail vein			
		[0–2]	[2–5]	[5–10]	>10
Experiment 1	Observer A (*n*)	7	2	2	1
	Observer B (*n*)	3	4	1	1
Experiment 2	Observer A (*n*)	11	3	5	0
	Observer B (*n*)	7	5	3	1

### Inter-observer variability for tail vein activity measurement

**Fig. 3:**
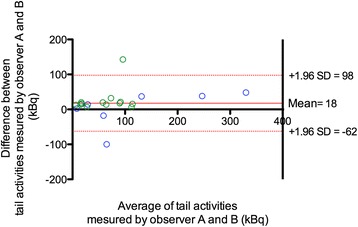
Relationship between quantitative values recorded by observers **a** and **b**. Tail vein activity measurements (kBq) were compared using Bland–Altman plot. The first experiment data is displayed in *blue* and the second experiment data in *green*

Bland–Altman analysis is shown in Fig. 3 and was conducted on all measurements of both experiments. This analysis demonstrated that the mean difference between tail vein activity measurements obtained by observer A and B was 18 kBq with narrow 95 % confidence intervals. Overall, there was a good inter-observer agreement when using qualitative analysis with a kappa value of 0.72 (95 % confidence interval (CI) 0.52–0.92) and a moderate agreement when using quantitative analysis with *ρ*_c_ value of 0.94 (95 % CI 0.88–0.97). Discordant results between observers occurred in 23.1 and 21.9 % of the first and second experiment SA-PET/CT examinations, respectively. In all but one case, observer A, indicated the presence of extravasation whereas observer B did not. Discordant results related to cases of low remaining activities in the tail, ranging from 0.04 to 0.73 % of theoretical injected activity.

As there was low inter-observer variability for tail vein activity measurement, the following results were assessed only for observer A, who identified more episodes of extravasation.

### Comparison between uncorrected and corrected data

**Fig. 4:**
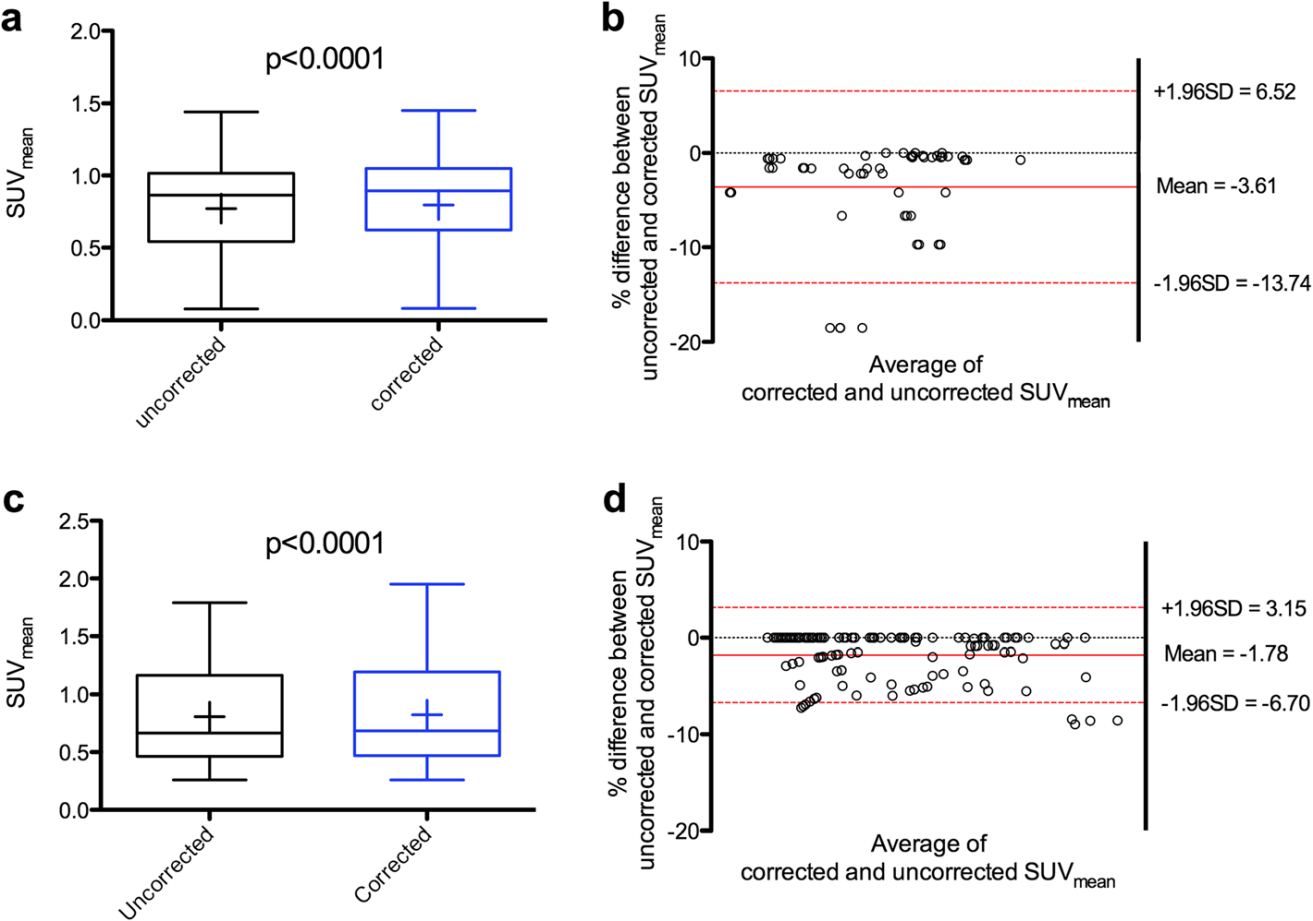
Comparison of corrected and uncorrected SUV_mean_ values. **a**, **b** Upper panels display the SUV_mean_ values of the first experiment, and **c**, **d** lower panels display the SUV_mean_ values of the second experiment, as recorded by observer A. Data is shown as Tukey boxplots (*left panels*, lines displaying median, 25th and 75th percentiles; *cross* represents the mean value) and Bland–Altman plots (*right panels*)

In each experiment, there was a significant difference between uncorrected and corrected SUV_mean_ values with *p* values <0.0001 (Fig. 4a, c). Bland–Altman analyses showed that mean percent differences between uncorrected and corrected SUV_mean_ values in the first and second experiments were −3.61 and −1.78, respectively (Fig. 4b, d).

### Impact of tail vein extravasation on assessment of therapeutic response

#### Molecular analysis

On day 3, immunohistochemistry studies showed decreases in p4E-BP1 phosphorylation, a downstream marker of mTOR activation in treated animals. On days 4 and 5, Phosphoinositide 3-kinase/mammalian target of rapamycin pathways exhibited partial recovery with almost complete recovery by day 7. When focusing on cell proliferation, Ki-67 staining was statistically and significantly lower in the treated group as compared with controls on days 3, 4, and 5. By day 7, there was no difference between control and treated groups. These results were detailed in another publication [16] and were used as the reference standard in the present work.

#### SA-PET analysis

**Fig. 5:**
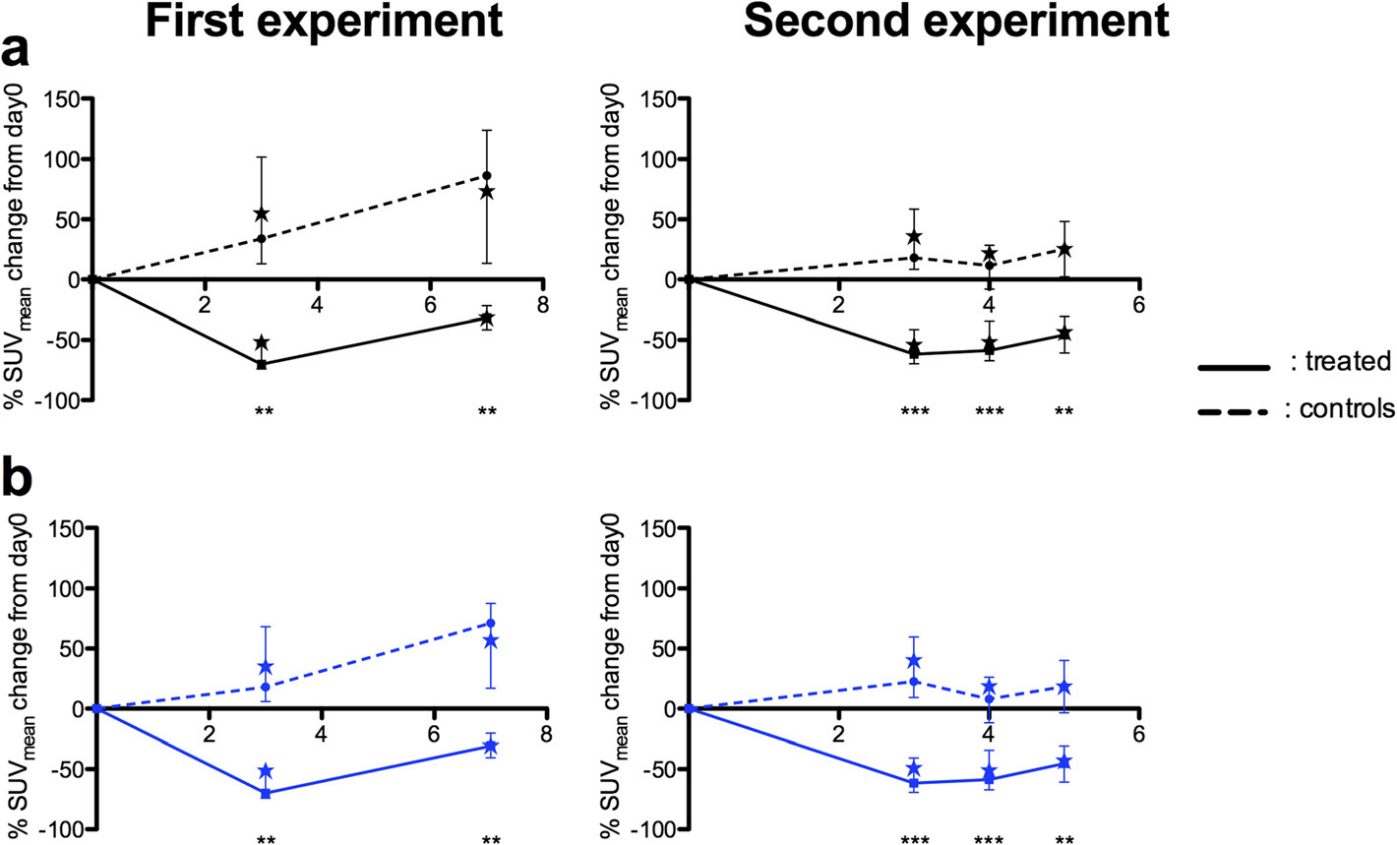
Uncorrected and corrected quantitative values in two therapy assessment experiments. Standard uptake values (SUV_mean_) relative to day 0 (medians, quartiles and means (*star*) for control and treated groups across all times points for the first and second experiments with uncorrected (**a**) and corrected (**b**) data. Quantitative values were extracted from observer A data. Legend for *p* values: ***p < 0.001, **p < 0.01, and *p < 0.05

Both corrected and uncorrected data showed significant decreases in ^18^F-FDG uptake on day 3 in treated groups in each experiment with a partial uptake recovery by day 5 (second experiment), which became more pronounced on day 7 (first experiment). In both experiments, significant differences in median %SUV_mean_ values between control and treated groups were observed across all times points, either with corrected or uncorrected data. It is also noteworthy that quartiles were almost similar with corrected and uncorrected data (Fig. 5).

## Discussion

Gaining venous access in animals can be challenging, even for experienced researchers, leading to tracer extravasation and potentially biased quantitative values. The present study evaluated whether data corrected for tail vein extravasation using a subtraction method performed better than uncorrected data in predicting therapy response in a large panel of animals in two therapeutic response assessment protocols. The main findings were that (1) tail vein extravasations occurred frequently (in at least in 50 % of cases as assessed by each observer across both experiments) and (2) that its frequency was not linked to treatment, time point within a given experiment, nor the cumulative number of injections. One would have expected the treatment-associated side effects, such as dehydration and cachexia, as well as repetitive injections, to impact on the quality of tail vein injections. Observed extravasation frequencies ranged from 50 to 92.3 % within the experiments described, which is much higher than those observed in other publications, reaching 40 % at worst [12, 13, 17]. However, it is likely that these authors did not look for extravasation with the same scrutiny. For example, in the study of Groman et al. [12], the presence of a misinjection was considered only when it exceeded 10 % of the injected dose (Table 2). This led to a more limited assessment of the problem by not accounting for the smallest extravasations. However, our results demonstrated that, despite one observer identifying extravasation more frequently, there was little inter-observer variability in quantifying the amount of activity extravasated using the tail vein correction process. These findings suggest that this technique is reproducible.

**Table 2:** Summary of the main studies that have evaluated tail vein extravasation

	Vines et al.	Chang et al.	Groman et al.
Animal model	Mice originally used for various oncology studies	Human HCT116 colon cancer xenografted nude mice (female, Charles River Laboratory)	Albino mice (males, 15 to 20 g, Swiss CD-1(ICR)BR, Charles River Laboratory)
Number of animals	50	12	30
Injection method	In-house needle catheters 30-gauge needles insert into 15 cm of polyethylene 10 tubing with a Blunt 30-gauge Luer-lock hub	29.5-gauge Terumo insulin syringe	Needle without precision
Tracer	^18^F-FDG	^18^F-FPP(RGD)_2_	Mixed of two reagents: ^99m^Tc-EB1 and colloid gold
Injected activity	2–9 MBq over 20–30 s	1.9–3.8 MBq	-
Injected volume	170 μL	100 μL	100 μL of mixture
Misinjection frequency and assessment	7/50 (14.0 %)	4/23 (17.4 %)	12/30 (40.0 %)
	Intermediate or poor injection based on qualitative assessment	Visual inspection	Injection efficiency <90 % based on quantitative assessment
Quantitative evaluation	Mean %ID/g on tail ranging from 2.4 to 28.4	Percentage-intended dose not injected ranging from 0.5 to 9.1 %	Percentage-intended dose not injected ranging from 12 to 63 %

Remaining activity in the tail was measured on in vivo imaging data because a cross-calibration between the SA-PET system and the dose calibrator was undertaken and because we had previously shown that ex vivo counting (taken as gold standards) and Inveon SA-PET/CT data was well correlated [18]. Moreover, Vines et al. had also previously shown an excellent correlation between in vivo imaging measurements and ex vivo gamma-well counter data [13]. Based on the SUV formulae, residual activity in the tail (equal to 16.9 % at worst in the present study) resulted in an underestimation of SUV values when uncorrected, as shown in Fig. 4. Chang et al. showed similar results [17]. In the current experiments, the molecular targeted therapy that was tested induced a marked decrease in ^18^F-FDG uptake. The median relative changes in the first and second experiments for treated animals were −70.02 and −61.69 %, respectively, using uncorrected data. Therefore, one could argue that the bias induced by tail vein extravasation would have a more pronounced impact when testing other therapies by inducing subtle changes in ^18^F-FDG uptakes. However, differences between control and treated groups were less important on days 4 to 7, when the therapy had been withdrawn, and there was still no difference between uncorrected and corrected data at these time points. Also, the situation of important extravasations, that could occur when junior researchers with little experience perform tail vein injection, or in the case of treatments inducing major cachexia, may require correction for extravasation.

The technique used in the present study was based on the assumption that there was no interstitial absorption of the tracer in the tail between the injection and the imaging session. Groman et al. [12] found that the tracer was retained at the injection site over an observation period of 7 days, but they used a 20-nm colloidal reagent tracer that had a higher steric hindrance than ^18^F-FDG. To the best of our knowledge, there are no available studies evaluating this phenomenon with ^18^F-FDG. Regarding the relatively short uptake period (<120 min), lymphatic clearance was considered negligible in the present study.

Finally, this study was performed in rats bearing tumors, whereas the most frequently used animal model in cancer research is the mouse. Given that we found that a percentage of injected activity remained in the tail (range 0.04–16.9 %) in the same or lower order of magnitude than in other studies on mice, we are confident that similar results would be expected with mice (Table 2).

## Conclusions

Despite the relatively high frequency of tail vein extravasation, the low activity remaining in the tail vein following extravasation (never higher than 20 % of the injected dose) led to a negligible magnitude of error and did not interfere with the cancer therapy assessment in rats bearing subcutaneous tumors undergoing targeted therapy. Assessment of therapeutic response using data uncorrected for tail vein extravasation provided similar results to those following correction. Nevertheless, there was no inter-observer variability when quantifying tail vein activity suggesting that the correction of data for tail vein extravasation can be reproducibly performed and may be important when assessing therapies inducing more subtle SUV changes between groups or when important extravasations occur.

## Ethics

The regional Ethics Committee granted approval to conduct this study (no. N/02-10-09/18/10-12). All procedures performed in this study involving animals were in accordance with the ethical standards of the regional research committee and with the 1964 Helsinki Declaration and its later amendments.
